# A Theory-Based Approach for Developing Interventions to Change Patient Behaviours: A Medication Adherence Example from Paediatric Secondary Care

**DOI:** 10.3390/healthcare3041228

**Published:** 2015-12-04

**Authors:** Gemma Heath, Richard Cooke, Elaine Cameron

**Affiliations:** 1 Department of Psychology, School of Life and Health Sciences, Aston University, Birmingham B4 7ET, UK; E-Mail: r.cooke@aston.ac.uk; 2 Manchester Centre for Health Psychology, University of Manchester, Coupland 1 Building, Coupland Street, Manchester M13 9PL, UK; E-Mail: elaine.cameron@manchester.ac.uk

**Keywords:** intervention, paediatrics, adherence, behaviour change, self-management

## Abstract

In this article we introduce a Health Psychology approach to changing patient behaviour, in order to demonstrate the value of Health Psychology professional practice as applied within healthcare settings. Health Psychologists are experts in understanding, predicting and changing health-related behaviours at the individual, group and population level. They combine psychological theory, research evidence and service-user views to design interventions to solve clinically relevant behavioural problems and improve health outcomes. We provide a pragmatic overview of a theory and evidence-based Intervention Mapping approach for developing, implementing and evaluating interventions to change health-related behaviour. An example of a real behaviour change intervention designed to improve medication adherence in an adolescent patient with poorly controlled asthma is described to illustrate the main stages of the intervention development process.

## 1. Introduction

Patient behaviours are central to the success of any treatment programme, and consequently to health outcomes. It is no surprise, therefore, that health professional training programmes increasingly teach student practitioners to understand the cultural, psychological and social factors affecting patients’ behaviours [1]. However, there is far less training in the methods needed to turn this understanding into action to help patients change detrimental behaviours. A commonly recommended approach is to offer patient education or advice. However, this is often insufficient for generating and maintaining behaviour change as patients’ health decisions are typically driven by multiple influencing factors, not merely lack of knowledge. Dissemination of alternative methods is therefore required to help health professionals to support patient behaviour change. 

Health Psychologists are experts in understanding and changing health behaviours in individuals, groups and populations. They combine psychological theory, research evidence and service-user views to design interventions to solve clinically relevant behavioural problems and improve health outcomes [2]. Health Psychologists are distinct from Clinical Psychologists in that Health Psychologists use their knowledge of psychology to understand the predictors, causes, and consequences of *physical* illness and health, and to influence health outcomes as well as healthcare systems and policies [3]. Health Psychology practitioner posts are not yet widely funded in the UK National Health Service (NHS), meaning that the majority of health professionals cannot access Health Psychology services or Health Psychologist-led training. Yet, the need to support patients in changing their behaviour remains. This article aims to provide a practical guide for practitioners and researchers looking to develop behaviour change programmes within healthcare settings.

### 1.1. An Introduction to Behaviour Change Interventions

The term “intervention” simply refers to any combination of strategies designed to produce desirable behavioural or health outcomes at the individual, group or population level. In the development and evaluation of complex (*i.e.*, multi-component) interventions, emphasis is placed on the use of theory [4] as evidence suggests that theoretically-informed interventions lead to better outcomes [5]. Theories help us to make sense of complex phenomena by providing tentative explanations for why and under what circumstances behaviours occur. Interventions can then target these factors. Using behaviour change theory to develop interventions also provides a way of understanding an intervention’s effectiveness, or lack thereof [6].

Within the context of behavioural research, interventions have often been based on whole individual theories such as the Health Belief Model [7] so that the theory can be tested and a contribution made to the theoretical evidence-base. However, in healthcare settings there is a need for interventions that address clinical problems to improve health outcomes, without needing to adhere to rigid theoretical frameworks. Consequently, new methods have been developed which take the behavioural problem as a starting point and use insights from different theories to assess, solve or prevent that problem [8].

One such method, Intervention Mapping (IM) [9], is a “problem-driven” approach which combines one or multiple theories, empirical evidence and new research to develop behaviour change interventions. This approach aligns with Medical Research Council guidance for the development and evaluation of complex interventions [4] and helps to solve the problem of identifying, selecting and applying suitable theories. The following example was developed using an approach guided by Intervention Mapping.

### 1.2. An Introduction to the Illustrative Example

The behaviour change intervention described here was developed, implemented and evaluated as part of a service improvement consultancy conducted by author EC [10]. Although informed consent was obtained from the patient and their parent, the work was not conducted as research and ethical approval was not sought. Consequently the focus of this article will be on the intervention development process, rather than the effectiveness of the intervention. However, descriptions of intervention mechanisms and outcomes will be provided where helpful.

The Health Psychologist in Training (EC) was contracted by a paediatric respiratory service to design an intervention to address medication non-adherence in adolescent patients with poorly controlled asthma. Many of these patients exhibited problems adhering to their prescribed preventive medication regimens. It was anticipated that improved adherence would prevent escalation of treatment to stronger and more costly medications, and reduce the risk of exacerbations and hospitalisation [11].

The intervention was developed for one individual patient with the intention that it would be refined and adapted for use with other patients following evaluation. This ensured that the intervention content could be tailored to each individual, and fit with the existing one-to-one service model. In other contexts group interventions may be feasible. Regardless of mode of delivery, the underlying principles of intervention design are the same, but an individualised approach allows greater tailoring of intervention content, while a group approach is less resource intensive. 

## 2. The Process of Intervention Development

An overview of the intervention development process is presented in Table 1. Each of the stages is discussed in detail below.

**Table 1:** Overview of the intervention development process.

Stages of Intervention Development	Brief Description of Each Stage
Conducting a needs assessment	Develop understanding of why and how the target population needs to change. Specify the health problem and it associated consequences and the target problem behaviour(s).
Identifying determinants of the target behaviour	Develop an understanding of what is influencing or causing the problematic behaviour. Try to identify factors leading to or inhibiting that behaviour.
Setting intervention objectives	Set goals for your intervention in terms of changing the target behaviour, behavioural determinants and associated health outcomes.
Selecting behaviour change techniques (BCTs)	Choose behaviour change techniques that can be used in your intervention programme to help achieve your intervention goals.
Developing practical plans	Select methods for delivering your intervention that will be acceptable and engaging for the target population. Consider how you will assess intervention effectiveness.
Reporting intervention outcomes	Communicate the effects of your intervention to relevant others (e.g., health practitioners, researchers, commissioners, patients).

### 2.1. Stage One: Conducting a Needs Assessment

The first stage involves justifying the need for an intervention by assessing why and how target recipients need to change. This means specifying three components: (1) who the intervention is intended to target; (2) the health problem; and (3) the problematic behaviour. 

Whether the intervention is intended for an individual or group, the recipients can be defined in terms of a “target population”, for example adolescents with asthma or adults with diabetes. The target population should be specified as explicitly as possible, as the population characteristics will influence the design, scope and feasibility of the intervention. In the clinical example the target population was defined as follows:

Adolescent patients (aged 12 to 16 years) receiving secondary outpatient care for asthma, who experience frequent symptoms despite being prescribed appropriate medications, who are not suspected to have refractory asthma, who are suspected or known to have poor adherence to medicines (e.g., taking inhalers infrequently or at the wrong dosage level), and who have no contraindications for receiving the intervention, such as social, psychological or communication issues.

This specification encompasses age range, clinical context, diagnosis, symptoms, and suspected behavioural issues. Other characteristics might include gender, ethnicity, time since diagnosis, prescribed medications and so on, depending on the objectives of the intervention. Specifying the target population can be achieved through discussion with clinicians and/or those who intend to deliver, use or commission the intervention. It is also important to consider individuals for whom a behaviour change intervention may not be helpful.

Next, an understanding of the health problem (e.g., poorly controlled asthma) must be elucidated in terms of the prevalence (e.g., percentage of target population experiencing the problem), patterns (e.g., differences related to sex, ethnicity, age) and consequences of the problem (e.g., hospital admissions), which justify the need for an intervention. A range of specific health outcomes should also be identified, which will inform the selection of intervention outcome measures later in the process. Both of these aspects can be generated through reviewing research literature and clinical guidelines, and by accessing clinical expertise. In the example, the following health outcomes were identified from the British Thoracic Society guidelines [12]:

“Control of asthmatic condition indicated by presence of daily symptoms (including coughing, wheezing, chest tightness and breathlessness), sleep disturbance, frequency of exacerbations, use of rescue medication, and the extent to which activities are limited”.

Other health outcomes might include health service utilisation, physiological functioning, body mass index, or quality of life. While accessing the literature and clinical expertise, it is also advisable to note how each of the health outcomes might be measured. The identified asthma outcomes were measured using self-report questionnaires, but other means such as patient self-report diaries, direct observation, dosage metering devices, and prescription refill records would all have been appropriate. 

Finally, the problematic behaviour which will be the main target of the intervention must be assessed and fully described. This involves reviewing literature on the prevalence of the behaviour within the target population, and consequences of the behaviour for health and other outcomes. The target behaviour should be broken down into behavioural components where possible. For instance, in the asthma example rather than describing the behaviour of interest merely as “taking asthma medications as prescribed”, specific elements of the behaviour were identified:

Taking medications as prescribed, including “preventer” inhalers, “rescue” inhalers, and any given tablets or other medicines; taking these at the prescribed dosage using the correct inhalation technique or consumption method; taking them at the required intervals and at the required times without missing any doses; and refilling prescriptions when necessary to ensure no doses are missed.

At the end of the needs assessment there should be a thorough documented understanding of the problem to be addressed, including a specification for whom the intervention is intended (target population), and overviews of the health issue (including prevalence, consequences, health outcomes, possible measures) and the target problem behaviour (including prevalence, consequences, behavioural components, and possible measures). 

In cases where the intervention is intended for a known patient or group, a personalised assessment can also be conducted through discussion with them and their key health professionals, and through checking health records. Discussion with the adolescent patient and asthma nurses in the clinical example revealed that frequency of symptoms and the need for reliever inhaler medication were particularly salient outcomes for them. Although the overall problem behaviour was “non-adherence to medication”, the behavioural component most relevant to this patient was missing doses of one particular tablet.

### 2.2. Stage Two: Identifying Determinants of the Target Behaviour

Having specified the problem behaviour, the next task is to identify factors that influence that behaviour. These behavioural ‘determinants’ will be targeted directly by intervention strategies as it is presumed that changing the influencing factors will consequently change behaviour. Identifying influencing factors will also inform which behaviour change techniques (BCTs) are likely to work and why.

#### 2.2.1. Determinants in the Target Population

To assess behavioural determinants in the target population a literature review can be conducted or an existing review identified. Where there is little existing evidence, new research can be carried out. Relevant literature includes studies exploring patients’ motives for the behaviour or their perceptions of factors influencing the behaviour; studies examining predictors of the behaviour; theory-testing studies; and existing intervention studies that describe associations between manipulated factors and behavioural outcomes. Data can also be collected on the effectiveness and acceptability of different intervention modalities (e.g., face-to-face, telephone support). For the asthma intervention a narrative review of research literature and clinical guidelines was conducted to establish factors influencing non-adherence in adolescent asthmatic patients. The review identified 28 determinants, which were grouped into nine categories (see Table 2).

**Table 2:** Determinants of adolescent asthma medication adherence identified in the literature.

**1. Beliefs and Attitudes**
Beliefs about asthma (e.g., cause, severity, controllability)
Beliefs about medicines
Attitude towards clinic visits
Attitude towards non-adherence (e.g., anticipated regret)
**2. Experiences**
Perceived impact of asthma and medicines on daily life
Experience of symptoms
Previous experience of adherence and management
Prior experience of consequences (e.g., hospitalization)
**3. Knowledge**
Knowledge and understanding of asthma and medicines
Knowledge of appropriate response in acute attacks
Recognising danger signals and symptoms
**4. Social Influences**
Peer group influences (e.g., feeling normal, embarrassed, perceived support)
Family factors (e.g., parent involvement in medicine-taking)
Relationships with healthcare professionals
**5. Motivations and Intention**
Motivation to manage asthma
Tolerance of current illness state
Prioritising asthma and treatment
Intention to take medications
Preference for alternative therapies
**6. Capability**
Skills to use the medication devices
Organisational and scheduling abilities
Self-efficacy (e.g., perceived ability, confidence)
Practical barriers (e.g., lost medicines)
**7. Self-Perceptions**
Self-identity
Perceived autonomy in taking medications
Feelings of responsibility for health
**8. Emotions and Psychological Wellbeing** (e.g., fear of asthma exacerbations)
**9. Forgetting and Confusion**

The results of a literature review may include findings regarding social, environmental, cultural and other factors in addition to psychological variables (e.g., beliefs about illness/treatment). All identified factors should be recorded as these may inform intervention development and interpretation of outcomes. However the main factors required for developing a behaviour change intervention are the psychological variables, as these are amenable to change.

#### 2.2.2. Individual Determinants

Behavioural determinants can be evaluated through clinical assessment with the individual patient or by conducting focus groups or surveys with groups. Questions should relate to the patient’s experience of performing the behaviour, their views about the health condition to which the behaviour relates, perceived barriers and facilitators of the behaviour, and their attitudes and beliefs about the behaviour, associated healthcare services, and health outcomes (see Table 3 for questions asked in the clinical example). Supplementary discussions with family members and healthcare providers may elucidate additional factors. Knowledge and skills related to the behaviour should also be assessed. As aforementioned, targeting knowledge and skills may be insufficient to change behaviour, but they are nonetheless prerequisites for successful behavioural performance.

**Table 3:** Example qualitative questions asked of the adolescent asthma patient to elicit personal determinants of non-adherence behaviour.

Example Qualitative Questions
- What is it like for you having asthma?
- How do you feel about having asthma?
- What bothers you most about having asthma?
- Tell me about the medicines you have to take for your asthma?
- How often do you miss a dose of your medicine?
- Why is it that you miss some doses?
- When are you most likely to miss a dose?
- What do you do if you miss a dose?
- What makes it difficult to take your medicine regularly?
- Do you ever miss a dose on purpose?
- What would make it easier for you to take your medicine regularly?

A clinical assessment with the patient could also include questionnaire measures of factors identified from the literature review. For example, during the needs assessment stage the adolescent asthma patient completed questionnaires measuring beliefs about medicines [13], beliefs about asthma [14], beliefs about the consequences of taking the medication [15], and confidence in managing the condition [16]. Questionnaires were selected based on evidence that beliefs about asthma, medications, and self-efficacy were important determinants of adolescents’ adherence to asthma medicines. They highlighted several sub-optimal patient responses which were then selected for targeting within the intervention. 

At the end of this stage a list of behavioural determinants should have been identified from the literature and any clinical assessments which are hypothesised to influence the behaviour (including psychological variables that are amenable to change).

#### 2.2.3. Organising Framework

When gathering information on behavioural determinants it can be helpful to organise identified factors using a theoretical framework. Useful behaviour change frameworks include the Capability, Opportunity, Motivation model of Behaviour (COM-B) [17] and the Theoretical Domains Framework (TDF) [18,19]. The COM-B model hypothesises that interaction between three core components causes the performance or non-performance of behaviour: physical and/or psychological capability; physical and/or social opportunity; and reflective and/or automatic motivation. For example, a patient could be highly motivated to take their medications, but due to chronic stress does not have the psychological capability to consistently remember to take them, or is only able to take them during break-times meaning they have limited opportunity to take them. The TDF expands on the COM-B model, synthesising 128 behavioural determinants taken from different theories into 14 overall domains including: knowledge; skills; identity; beliefs about capabilities; optimism; beliefs about consequences; reinforcement; intentions; goals; memory, attention and decision processes; environmental context and resources; social influences; emotions; and behavioural regulation [19]. 

### 2.3. Stage Three: Setting Behaviour Change Intervention Objectives

The first two stages in the intervention development process provide a thorough knowledge base on which to establish the intervention. In this third stage the aim is to describe the objectives of the intervention in terms of changes in health outcomes, behavioural changes, and changes in psychological factors (the targeted behavioural determinants). 

#### 2.3.1. Health and Behaviour Change Objectives

From the work conducted in the needs assessment, appropriate health and behaviour outcome objectives can be selected. These outcomes will be measured before and after the intervention is delivered to assess its effectiveness. They must therefore be measurable, achievable and relevant for the patient. Working in collaboration with the patient and their healthcare provider can help to achieve this. Objectives should also be specific in their description, including the direction and magnitude of the intended change. In the clinical example, health and behaviour objectives were:

- Health objective: To achieve a decrease in general asthma symptoms (coughing, wheezing, chest tightness, breathlessness) from 1 to 2 days per week to no days per week.
- Behavioural objective: To achieve a decrease in the frequency of missed doses of (named medication) from once per week to no times per week.

#### 2.3.2. Behavioural Determinant Change Objectives

Next, from the identified behavioural determinants the intervention developers must select and justify those to target within the intervention. Which ones and how many are a matter for consideration. For some there will be greater evidence of a stronger connection with the behaviour; some will be more clearly relevant to the patient; some will be more feasible to change; and some will have clearer existing links with BCTs. It may be impractical to target all identified determinants, and also ill-advised as the resulting intervention might be difficult to evaluate. In some cases there may be one obvious determinant that is clearly and strongly causally linked to the behaviour, meaning the selection of a single determinant to target in the intervention is appropriate. However, generally interventions containing more than one strategy offer greater opportunity for inducing change [20]. 

#### 2.3.3. Mapping Determinants to Theory

This stage also involves mapping the identified empirical determinants to theoretical constructs. This means translating the determinants into components of behavioural theories or into stand-alone theoretical concepts that can be measured and tested for their ability to predict or explain behaviour. One benefit of doing this is that theoretical constructs are more “testable” than descriptive determinants, as they often have specific definitions and existing validated measures. The intervention outcomes can also be more easily applied in other contexts if well-defined constructs are used. Furthermore, the selection of BCTs in the next stage may be easier in relation to theoretical constructs, as some theories explicitly recommend certain techniques. 

This translational process is one part of the intervention development process that requires the most psychological expertise, as methods for interpreting empirical determinants as theoretical constructs rely on the intervention developers’ knowledge of behavioural theories. However, mapping determinants to the Theoretical Domains Framework [19] is also possible. An example of this in relation to non-adherence might be translating the empirical determinant, “belief that medicine causes weight gain” into the theoretical construct “physical outcome expectation” from Social Cognitive Theory [21] or “outcome expectancies” in the domain “beliefs about consequences” from the TDF [19]. In cases where a behavioural determinant cannot be translated into a theoretical construct (e.g., knowledge), it should still be included, but as an empirical rather than theoretical factor.

In the clinical example, two theories were identified as potentially useful for measuring and changing behavioural determinants. First, the extended Common-Sense Self-Regulation Model [22,23], which encompasses beliefs about the illness and medications. Second, Social Cognitive Theory [21], which encompasses self-efficacy (confidence in one’s own ability to perform the behaviour) and outcome expectations. Other determinants remained as stand-alone constructs, such as knowledge about asthma.

**Figure 1:**
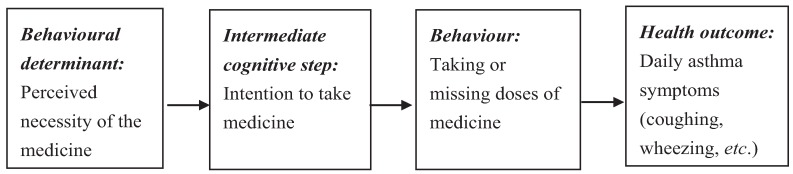
Explanatory model of adherence behavior.

The selected theoretical constructs can then be used as the basis for a simple explanatory model describing the mechanisms by which behaviour and health outcomes are influenced by the determinants. A simplified section of the explanatory model generated in the clinical example is shown in Figure 1. Note that in accordance with behavioural theories, determinants may influence behaviours directly or via the patient’s *intention* to perform the behaviour.

The final list of behavioural determinants, in the form of theoretical constructs and untranslated empirical constructs, should then be stated explicitly as “change objectives” [9]. For instance:

Determinant change objective: The patient will demonstrate an increase in perceived necessity of (named medication) for maintaining health and reducing symptoms (measured by the Beliefs about Medicines Questionnaire).

### 2.4. Stage Four: Selecting Behaviour Change Techniques

Having developed a model of determinants of the behaviour and specified change objectives for the intervention, the next phase involves linking behavioural determinants to a set of BCTs thought to be effective for eliciting/changing the target behaviours. That is, techniques that will bring about change to behavioural determinants and achieve the change objectives [24]. A BCT can be defined as an “active ingredient” of an intervention, or “an observable, replicable, and irreducible component of an intervention designed to alter or redirect causal processes that regulate behaviour” [25].

The components of some theories (e.g., self-efficacy from Social Cognitive Theory) already recommend specific techniques for changing behaviour, such as modelling and verbal persuasion [26]. For the remainder, Michie *et al.* (2013) have devised a categorised list of 93 BCTs which have been mapped to theoretical constructs using a consensus method [25,27,28]. These resources were used in the asthma example to select BCTs. The technique “information about health consequences” from the 93-item BCT taxonomy (Michie *et al.*, 2013) [25] (BCT 5.1) was selected to target the patient’s perceived necessity of their medicine, while another technique “prompts/cues” (BCT 7.1) was used to target the patient’s remembering to take the medicine. Every determinant to be targeted should be addressed by at least one BCT, and all BCTs should be intended to influence at least one determinant. There should be no unmatched determinants or BCTs.

### 2.5. Stage Five: Developing Practical Plans

Having identified potentially useful BCTs (e.g., goal setting, coping skills), these need to be translated into a practical intervention programme, outlining the timelines, measures, methods, materials and personnel to be used to deliver and evaluate the intervention. Interventions can be implemented in a variety of ways, for example, face-to-face, via telephone, using text messaging or mobile phone apps or paper-based methods, on a one-to-one basis or as group sessions. They can also be delivered by a range of health professionals (e.g., nurses, physiotherapists, psychologists) or trained “lay” facilitators. It is recommended that the intervention delivery information is compiled in a comprehensive manual that can be used by subsequent intervention deliverers. When it comes to delivering the intervention, practical plans should be implemented as closely as possible to those documented in the intervention manual. Any deviations should be recorded and taken into account when evaluating the intervention outcomes.

Components of the clinical example intervention were developed into practical plans with input from the asthma nursing team. These plans took into account the time and resource demands of usual healthcare practice. The intervention was therefore planned as three sessions, delivered with the patient over a four week period; one session each for the baseline assessment, delivery of BCTs, and evaluation. Sessions were designed to be one hour long, delivered one-to-one at either the patient’s home or in the hospital outpatient department. To deliver the BCTs the following methods were used: workbooks providing information, diagrams and links to online videos about asthma and the effects of medications, (BCT 5.1 “Information about health consequences”); and worksheets to facilitate the patient devising their own prompts for remembering the behaviour (BCT 7.1 “Prompts/cues”).

#### Planning an Evaluation

Evaluation involves employing methods to discover whether the intervention worked, how well it worked and how it worked. Methods of evaluation should be considered at the intervention development stage [6] and should include measures of process, effect and acceptability. 

Intervention effectiveness and outcomes can be measured by assessing changes to the behaviour and health outcomes using validated measures wherever possible. It is also important to measure the proposed mechanisms of action, that is, the psychological constructs that were targeted in the intervention (e.g., self-efficacy, illness perceptions) as this reveals which factors mediated the intervention effects. In other words, how the intervention worked and why it worked (or didn’t work). This is important as it forms the basis for future interventions [29]. Where existing questionnaires are not available, measures can be developed or adapted specifically for the intervention. For example, questions about knowledge of the condition may be developed using patient information leaflets.

In the asthma example, intervention effectiveness was evaluated using questionnaire measures and qualitative questioning conducted at baseline and in the final evaluation session. Measures were also taken at the end of the BCT delivery session, so that changes could be tracked across the whole programme. Measures included a questionnaire on current asthma symptoms to assess health outcomes (adapted from Asthma UK symptom diaries, 2012), the Medication Adherence Report Scale to assess behavioural outcomes [30], the Brief Illness Perception Questionnaire [14], the Beliefs about Medicines Questionnaire [13], the Child Self-Efficacy Asthma Self-Management Scale [16], and outcome expectancy scales developed according to guidance by Luszczynska and Schwarzer [15] to assess changes in determinants. The intervention was also evaluated using patient and parent feedback via programme evaluation forms, asking questions regarding how useful, enjoyable, and difficult they found the intervention.

### 2.6. Stage Six: Reporting the Intervention 

A clear and thorough description of the intervention should be provided to enable replication by other clinicians or researchers, and to facilitate synthesis of findings in any evidence reviews [25,31]. Under-reporting of intervention content and using variable terminology to describe BCTs has been highlighted as a weakness of previous intervention studies [4]. However the development and introduction of tools such as the Behaviour Change Technique taxonomy [25,31] provide a common language for describing the “active ingredients” of interventions in sufficient detail. Guidelines for reporting behavioural interventions have also been developed, including a Template for Intervention Description and Replication (TIDieR) [32] and Standards for Quality Improvement Reporting Excellence (SQUIRE) [33].

When reporting the outcomes of interventions developed for research purposes, the aim may be to comment on statistically significant changes in determinants, behaviours and health outcomes, and the generalisability of the findings to the wider patient population, in which case appropriate statistical methods should be used. However, for interventions developed in clinical practice, outcomes could be usefully reported in patient notes. In such circumstances, changes in responses to the qualitative questions and questionnaire measures should be carefully considered and reported in terms of both direction and magnitude of change.

## 3. Discussion and Conclusions

In this article we describe an approach to developing behaviour change interventions, with a clinical example designed to improve adherence to treatment in adolescents with poorly controlled asthma, which was guided by Intervention Mapping [9]. This systematic framework offers a transparent, problem-solving approach to address health needs, using theory, research evidence and service-user perspectives to tailor the intervention to the target behaviour and population. 

From the early stages of intervention development, we were able to identify psychological factors amenable to change in the target population that were linked with the target behaviour (adherence to treatment). Eliciting information directly from the patient enabled the selection of intervention objectives that directly addressed the patient’s individual needs. A mapping process was then used to interpret empirical determinants as theoretical constructs, from which appropriate BCTs were selected and implemented. The intervention was evaluated in terms of process and effect using qualitative and quantitative methods. 

There are several characteristics of this approach that should be considered by would-be intervention developers. The first is the explicit involvement of patients and healthcare providers in selecting behavioural targets, psychological determinants and BCTs. Developing a shared understanding of the behavioural problem with the patient, prioritising patient-identified behaviours and beliefs and agreeing on intervention objectives builds confidence, skills and motivation to implement and maintain behavioural changes, and so increases the likelihood of intervention effectiveness. However, this may comprise a limitation if engagement of these parties is low. Consequently, intervention developers may need to devise strategies for increasing service-user and health professional involvement. 

A second consideration is the time and resources required to plan, implement and evaluate the intervention. The flexibility of this approach means that it is suitable for both resource-rich and resource-poor environments. In resource-rich environments, full literature reviews and comprehensive individual assessments can be conducted. In resource-poor environments however, the method can be adapted. Greater reliance can be placed on clinical expertise to inform understanding of the health and behavioural issues; determinants may be identified from existing reviews and clinical guidelines, supplemented by patient reports; and determinants can be directly mapped to an overarching theoretical framework rather than individual theories. 

This case study demonstrates the feasibility of developing evidence and theory-based interventions to change patient behaviour within healthcare settings. The empirical and theoretical basis of this approach means that interventions have a greater chance of being effective, and the reasons for these effects can be deduced. Consequently, interventions can be refined and adapted iteratively to meet the needs of subsequent patients. The principles and processes of a Health Psychology approach to intervention design are comprehensively described so that lessons can be drawn by health professionals working to address behavioural challenges in various clinical populations.
